# The interactive effects of mowing and N addition did not weaken soil net N mineralization rates in semi-arid grassland of Northern China

**DOI:** 10.1038/s41598-019-49787-6

**Published:** 2019-09-17

**Authors:** Yükun Luo, Changhui Wang, Yan Shen, Wei Sun, Kuanhu Dong

**Affiliations:** 1 College of Animal Science and Technology, Shanxi Agricultural University, Taigu, Shanxi 030801 China; 2 State Key Laboratory of Vegetation and Environmental Change, Institute of Botany, Chinese Academy of Sciences, Beijing, 100093 China; 3 Key Laboratory of Vegetation Ecology, Ministry of Education, Institute of Grassland Science, Northeast Normal University, Changchun, Jilin, 130024 China

**Keywords:** Element cycles, Grassland ecology

## Abstract

As the largest portion of the terrestrial ecosystems, the arid and semi-arid grassland ecosystem is relatively sensitive and vulnerable to nitrogen (N) deposition. Mowing, the main management in Inner Mongolia grassland also has deep direct and indirect effect on N transformation by removing the nutrient from soils. However, the interaction effect of N addition and mowing on N transformation is still unclear, especially in semi-arid grassland. Here, we conducted a field-manipulated experiment to assess N addition (10 g N m^−2^ y^−1^) and mowing (in the middle of August) effects on soil net N mineralization rate across 4 growing seasons (2006–2009) in a semi-arid grassland in Inner Mongolia of northern China. We found that N addition with or without mowing led to significant effect on soil ammonification rate and net N mineralization rate, but had no significant effect on nitrification rates. Furthermore, mowing had no significant effect on soil net N mineralization, ammonification and nitrification rates. N addition and Mowing decreased microbial respiration and metabolic quotient, whereas the interaction of N addition and mowing had no significant effect on microbial respiration and metabolic quotient. Our results indicated that the effects of mowing and N addition did not interactively weaken soil net N mineralization rates in a semi-arid grassland of Northern China. Therefore, the anthropic management (i.e. mowing for hay once a year) with N addition may be a sustainable approach for restoration and reconstruction of vegetation in the abandoned grassland  of Northern China.

## Introduction

Atmospheric nitrogen (N) deposition is one of the main nitric sources of the terrestrial ecosystem, and might aggravate under the influence of human disturbance and global climate change. Recent research has demonstrated that due to the continuous increasing of NO_3_^−^ deposition and the significant decrease of wet NH_4_^+^ deposition, the total N deposition has changed from a rapid increase towards stability from 1980 to 2015 in China^1^. Meanwhile, N deposition has been shifted from being wet dominated to almost equal contributions of dry and wet deposition because of the increased atmospheric dry deposition and the decreased wet deposition^1^. Nitrogen addition has been recognized as an essential nutrient for plant production and microbial activities in terrestrial ecosystems, but it is mostly limited in terrestrial ecosystems^2–6^. There is mounting evidence for N fertilizer addition increased soil inorganic nitrogen content, but we know little about its effects on soil N transformation in semi-arid grassland. Although more and more studies have been conducted to report the impact of grassland management (i.e. mowing and N addition) on the structure and function of grassland ecosystems. Compared with other management, the research on the effect of mowing on grassland N transformation is a little, and the results are different, particularly the interactive effects of mowing and N addition on soil net N mineralization is also not clear.

Most of previous studies on the responses of soil N mineralization to N addition focused on soil physical and chemical characteristics, the frequency of N application and addition time, the amount of N addition and N fertilizer types^7,8^. Some researchers reported that N addition were promoting effects on the content of ammonium N, nitrate nitrogen and available nitrogen, and increased the microbial activity involved in soil N transformation, N transformation in typical and alpine grassland and wetland^9,10^. Normally grassland ecosystems are limited by N, appropriate rate of N addition increased the soil inorganic N content and organic matter content, alleviated the competition between plants and soil microbe, then promoted microbial N transformation. However, high N application rate inhibited microbial activity, and then reduced the N transformation rates. For example, there were also a few studies holding that lower N addition significantly increased soil net N mineralization rates, while higher N addition had opposite results in typical grassland of northern China. The inconsistent findings reported in previous studies might result from the impacts of experimental duration and other factors on the N addition responses of ecosystem N transformation. For example, N addition increased net N mineralization rate with the increasing N addition in a short time^11,12^. However, long-term N application decreased soil N mineralization rate, due to the decreased pH and decreased microbial activity and diversity^13^. The main reason is that long-term N addition lead to soil acidification, and increased the content of refractory organic matter and toxic substances in the soil, thus inhibiting soil microbial activity and structural diversity, thereby reducing soil N transformation. Furthermore, other environmental factors can further compromise N addition effects on ecosystem N transformation. Ecosystems may change toward P limitation as a consequence of ameliorated N limitation^14^, which will further restrain the N effect. All these factors contribute to the variation in ecosystem responses to N addition. Therefore, it is necessary to explore interactive effects of N addition and other factors on soil N transformation.

Mowing for harvesting hay is the most important ways in grassland management. The impacts of mowing on soil N transformation have been low documented and the results are inconsistent in different studies^15,16^. Mowing in grasslands may affect soil N transformation by changing soil microclimate (Soil temperature and water content). For instance, most of studies reported that long-term mowing increased soil temperature, decreases soil water content, which significantly affected soil microbial activity and diversity, then affect N transformation^11,17–20^. Some other studies showed that mowing increased plant root exudates and soil microbial biomass, reduced the C/N ratio of microbial biomass, then improved N transformation^20,21^. The loss of C substrate supply from photosynthesis and aboveground litter associated with mowing could reduce soil N transformation^22^. Successive and intensity mowing must lead the lost and unbalance of substance and energy, and lead up to degradation succession of the grassland^2^. The mechanism of mowing effects on soil N transformation is controlled by the complex interaction of changing soil microclimate and the availability of C substrate for root and microorganisms, and varies with vegetation type and soil texture. Therefore, more detailed studies in different ecosystems are needed.

The main effects of N addition and mowing on soil N transformation on natural and artificial grassland were rarely reported, especially in abandoned grassland in northern China. Meanwhile, examination of the possible interactions between N addition and mowing on soil N transformation in semiarid grasslands has received little attention. The effect of mowing on soil N transformation might interact with N addition since mowing decreased soil carbon, while N addition increased N supply and enhanced plant nutrient uptake, amplifying the limitation of carbon availability^23^. Here, we hypothesized that N addition and mowing have a compensatory effect on nutrient cycling in N limited grassland of northern China. To investigate effects of mowing, N addition and their potential interactions on soil N mineralization, we conducted an N addition and mowing experiment in an abandoned grassland in Northern China through 2006 to 2009.

## Materials and Methods

### Site description

This study site was performed in the Restoration Ecological Research Station of Institute of Botany (42°02′N and 116°17′ E), Chinese Academy of Sciences (IBCAS), near Dunlun county, in Inner Mongolia, Northern China. The mean annual temperature is 2.1 °C, with mean monthly temperatures ranging from −17.5 °C in January to 18.9 °C in July. The mean annual soil temperature (0–5 cm depth) is 5.3 °C, ranged from minimum in January (−16.7 °C) to maximum in July (24.3 °C), with mean soil temperature from 14.9 to 24.3 °C during the growing seasons (May-September). Mean annual precipitation is 385.5 mm, almost >86% of which occurs in growing season (May – September), and the mean annual evaporation is 1748 mm. These data came from a long-term observation (1953–2007) at Restoration Ecological Research Station of Institute of Botany. The soil of the study site was classified as chestnut soil. The dominated vegetation is *Artemisia frigida*. Traditional land uses include livestock grazing and farming. Soil and plant characteristics were measured before start of the experiment in August of 2005 and reported by Wang *et al*.^17^. Duolun Country located in the ecotone between grassland and the dryland agriculture region in Northern China is an ecological fragile zone. In our study area, large parts of the natural steppe were converted to cropland in the 1960s. As a restoration measure, about 80% cropland was abandoned in 1995 and either remained without any further management or has been used for hay production or grazing^17^.

### Experimental design

The experiment was conducted a randomized complete block design with twenty-four 4 m × 4 m plots, the distance between plots was 2 m. The four different treatments in the experiment were control (C), N addition (N), mowing (M) and N addition plus mowing (N + M) with six replicates. 10 g N m^−2^ yr^−1^ was added in the middle of growing season from 2006 to 2009. N fertilizer (as NH_4_NO_3_) was applied before the rains. Mowing was carried out in the middle of August in 2006–2009, leaving 3 cm of stubble, and moved away all plant material from the mowing plots. Details of the experiment design reported by Wang *et al*.^17^.

### Sampling and measurements

Soil samples were collected in the middle of August in 2006–2009 when the most vigorous microbial activity occurred in this area. In order to avoid spatial heterogeneity, 5 cores (4.5 cm in diameter and 12 cm in depth) were collected from 0–10 cm depth in each plot at random locations, and then completely mixed into one composite fresh sample. Then plant roots, litter and large stones were removed by sieving (sieve mesh 2 mm), the soil samples were packed in bags in ice blocks and transported to the laboratory. Each sample was divided into two sub-samples and stored in the fridge at 4 °C. The first part for tests microbial biomass carbon (MBC) and microbial biomass nitrogen (MBN), the second part for tests potential microbial respiration (MR).

Microbial biomass was measured using the fumigation-extraction method^24^. Firstly, fumigated 20 g fresh soil samples for 24 h with ethanol-free CHCl_3_. Secondly, soil extracts from the fumigated and controlled samples were obtained by shaking soil samples with 50 ml of 0.5 M K_2_SO_4_ for 1 h, the supernatant was filtered using a 0.45 mm membrane filter. Finally, filtrate was measured using an automated TOC/TN analyzer (Analytik Jena AG, Jena, Germany) to determine extractable C and N. MBC and MBN were calculated from the difference between extractable C and N contents in the fumigated and non- fumigated samples using conversion factors (*k*_EC_ and *k*_EN_) equal to 0.38 and 0.45, respectively^25^.

MR was measured by alkali absorption of CO_2_ evolved at 25 °C and optimal soil moisture for 1 week followed by titrating the residual OH^−^ with a standardized acid^26^. Briefly, 25 g fresh soil was incubated in a 500 ml glass flask. The glass flask was connected with a glass tube in which 5 ml of 0.05 M NaOH solution was injected to capture the CO_2_ produced by the soil microbes. The metabolic quotient (*q*CO_2_) was calculated from the estimates of MR and MBC^27^.

At August in 2006–2009 in each plot, two polyvinyl chloride tubes (4.5 cm in diameter and 12 cm in height) were hammered into the soil to a depth of 10 cm. The floor litter was removed before sampling. One of the two tubes from each subplot was retrieved and brought the soil in the tube back to the laboratory. The other tube with a breathable and impermeable sealing film on the top was incubated *in situ* for 30 days before being retrieved. Ammonification rate (R_amm_), nitrification rate (R_nit_) and net N mineralization rate (R_min_) were calculated from differences in soil NH_4_^+^-N and NO_3_^−^-N concentrations between the initial soil sample and the incubated sample. To analyze soil inorganic N concentrations, a 10 g fresh soil samples was taken from each sieved soil sample, and extracted with 50 ml of 0.5 M K_2_SO_4_ solution. The soil suspension was rigorously shaken for 1 h in a reciprocal shaker and then filtered through filter paper. Soil solutions were immediately analyzed for NH_4_^+^-N and NO_3_^−^-N on a FIAstar 5000 Analyzer (Foss Tecator, Denmark).

Soil temperature (ST) at a depth of 10 cm was measured between 9:00–10:00 am twice at August in each plot using a soil thermometer (Longstem Thermometer 6310, Spectrum Technologies, Plainfield, USA). Soil water content (SWC, 0–10 cm) was measured in each plot gravimetrically during the sampling period. The incubation tests were conducted in the laboratory of Institute of Botany, Chinese Academy of Sciences. All results were expressed on an oven-dried soil basis (105 °C, 24 h).

### Statistical analysis

Statistical analyses focused on the data of August in 2006–2009. Three-way ANOVA was used to determine the effects of year, N, M and N + M on net N mineralization rate (R_amm_, R_nit_, R_min_), microbial biomass (MBC, MBN, MBC/MBN) and microbial respiration (MR, *q*CO_2_). The responses of SWC and ST, net N mineralization rate, microbial biomass and microbial respiration to N or M or N + M were performed with two-way ANOVA. Unless otherwise stated, the differences were considered statistically significant at *P* < 0.05. Simple and multiple linear and nonlinear regression analyses were used to examine the relationships between net N mineralization rate, microbial respiration and SWC, ST, microbial biomass. All statistical analyses were conducted using SPSS 23.0 (IBM SPSS, Inc., Chicago, Illinois, USA).

## Results

### Soil water content and temperature

**Figure 1:**
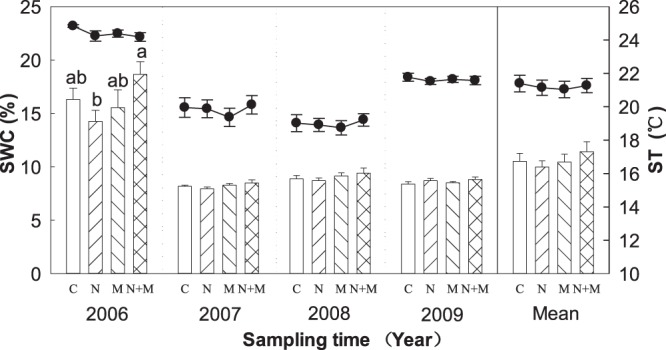
Effects of N addition and mowing on soil water content (SWC, bars) and temperature (ST, lines). Data are represented as the means ± SE (standard error). Significant differences are denoted by different letters (*P* < 0.05). C: control; N: nitrogen addition; M: mowing; N + M: nitrogen addition plus mowing.

Significant interannual variations of precipitation (387.2 mm, 185.6 mm, 318 mm and 172.1 mm from 2006 to 2009, respectively) in the growing season (August) were observed in this study site^28^. Meanwhile, there were significant interannual variations in both soil temperature (ST) and water content (SWC) from 2006 to 2009 (Table 1). ST and SWC in 2006 were significantly greater than that in 2007–2009 (Fig. 1; Table 1). N, M and N + M treatments had no significant effect on ST and SWC from 2006 to 2009 (Fig. 1). However, mean SWC in N + M treatment was significantly higher than that in N treatment only in 2006 (Fig. 1). In average, the main effect of N, M and N + M treatments had no significant effect on ST and SWC across 4 years (Fig. 1).

**Table 1:** Results (F-values) of repeated-measures ANOVAs for the effects of year (Y), mowing (M), N addition (N) and their interactions (Y × M, Y × N, M × N and Y × M × N) on soil temperature (ST), soil water content (SWC), ammonification rate (R_amm_), nitrification rate (R_nit_), net N mineralization rate (R_min_), microbial biomass carbon (MBC), microbial biomass nitrogen (MBN), microbial respiration (MR) and metabolic quotient (*q*CO_2_).

Source	ST	SWC	R_amm_	R_nit_	R_min_	MBC	MBN	MBC/MBN	MR	qCO_2_
Y	156.01***	125.29***	7.08***	21.71***	12.79***	3.09***	34.90***	14.99***	27.79***	7.27***
M	0.35	4.28*	1.18	1.27	1.65	0.42	1.31	2.48	5.63*	1.87
Y × M	0.10	1.32	2.82*	1.91	3.92*	0.65	0.59	1.39	7.52***	3.18*
N	0.00	0.43	6.83*	1.43	8.83**	1.52	12.19***	1.86	0.14	1.20
Y × N	0.75	0.14	0.92	7.59***	0.82	0.49	7.44***	14.01***	0.17	0.26
M × N	1.64	5.08*	0.69	0.88	1.49	3.19	0.62	2.56	7.06**	14.96***
Y × M × N	0.13	3.35*	0.35	2.68*	1.05	1.24	0.39	0.23	3.37*	4.62**

Significance levels: *P < 0.05; **P < 0.01; ***P < 0.001.

### N addition and mowing effects on soil microbial biomass

**Figure 2:**
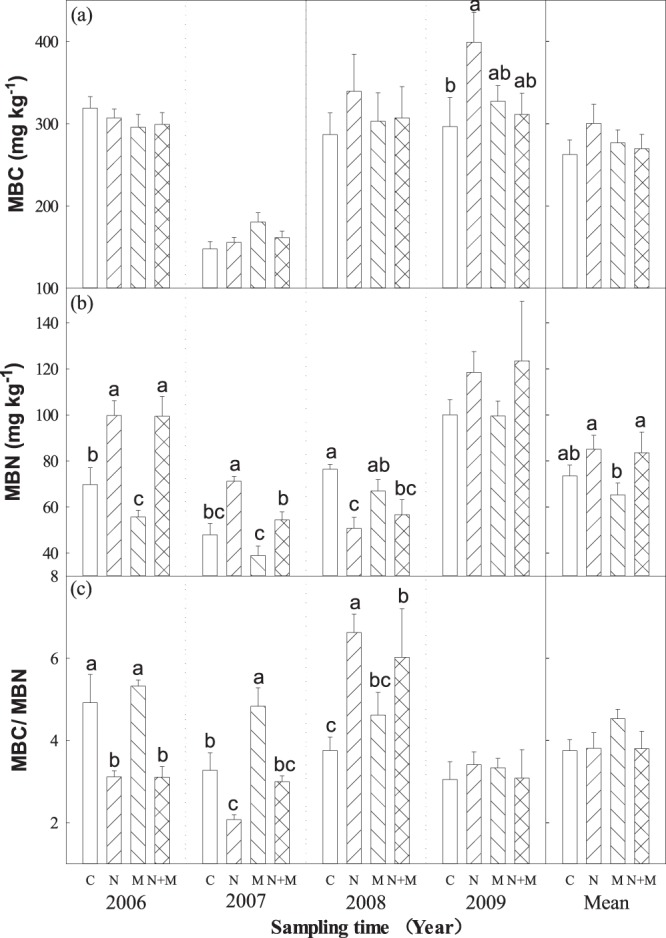
Effects of N addition and mowing on soil microbial biomass carbon (MBC) and microbial biomass nitrogen (MBN). Data are represented as the means ± SE (standard error). Significant differences are denoted by different letters (*P* < 0.05). C: control; N: nitrogen addition; M: mowing; N + M: nitrogen addition plus mowing.

Driven by the large variability amounts of moisture and temperature in the growing period across 4 year from 2006–2009, significant interannual variation in all of the microbial biomass carbon (MBC), microbial biomass nitrogen (MBN) and the ratio of microbial biomass carbon and nitrogen (MBC/MBN) were observed (Table 1). MBC was significantly lower in 2007 than that in 2006, 2008 and 2009 (Fig. 2a; Table 1). N, M and N + M treatments had no significant effect on soil MBC in 2006–2008 (Fig. 2a). However, MBC in N treatment was significantly greater than M and N + M treatments, which were significantly greater than C treatment in 2009 (Fig. 2a). In average, the main effect of N, M and N + M treatments had no significant effect on soil MBC across 4 years (Fig. 2a).

The effects of N, M and N + M treatments on soil MBN were inconsistent across 4 years (Fig. 2b). Soil MBN in N and N + M treatments were greater than that in M and C treatments in 2006 and 2007 (Fig. 2b). Soil MBN in C and M treatments were greater than N and N + M treatments in 2008 (Fig. 2b). In addition, soil MBN in N and N + M treatments were slightly greater than that in C and M treatments in 2009 (Fig. 2b). In average, soil MBN in N and N + M treatments were slightly greater than C and M treatments across 4 years (Fig. 2b).

N, M and N + M treatments significantly affected on soil MBC and MBN, then resulted in significant effect on the ratio of MBC/MBN (Fig. 2c). The ratio of MBC/MBN in C and M treatments were greater than N and N + M treatments in 2006 and 2007 (Fig. 2c). However, the ratio of MBC/MBN in N and N + M treatments were greater than C and M treatments in 2008 (Fig. 2c). In addition, N, M and N + M treatments had no significant effect in the ratio of  MBC/MBN in 2009 (Fig. 2c). The results showed that the ratio of bacteria to fungi tend to be stable in temporal scale. In average, N, M and N + M treatments had no significant effect on the ratio of MBC/MBN across 4 years (Fig. 2c).

**Figure 3:**
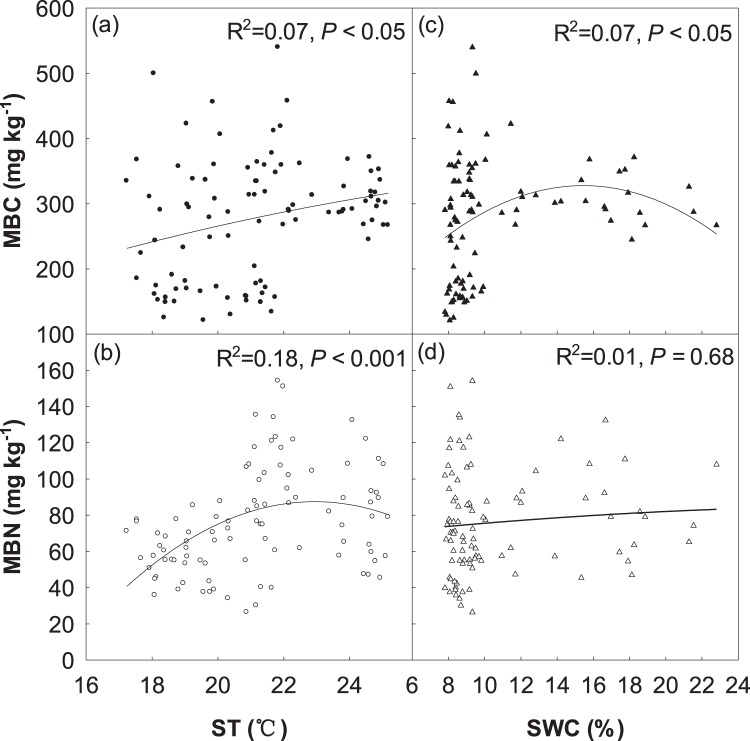
Relationships of soil microbial biomass carbon (MBC) and microbial biomass nitrogen (MBN) with soil temperature (ST) and soil water content (SWC).

Moreover, we found that N, M and N + M treatments induced changes in soil microbial biomass were closely associated with ST and SWC (Fig. 3). Interestingly, MBC increased significantly with ST (R^2^ = 0.07, *P* < 0.05; Fig. 3a) and SWC (R^2^ = 0.07, *P* < 0.05; Fig. 3c), respectively. Meanwhile, MBN increased significantly with ST (R^2^ = 0.18, *P* < 0.001; Fig. 3b), but had no significant relationship with SWC (Fig. 3d).

### N addition and mowing effect on soil microbial respiration

**Figure 4:**
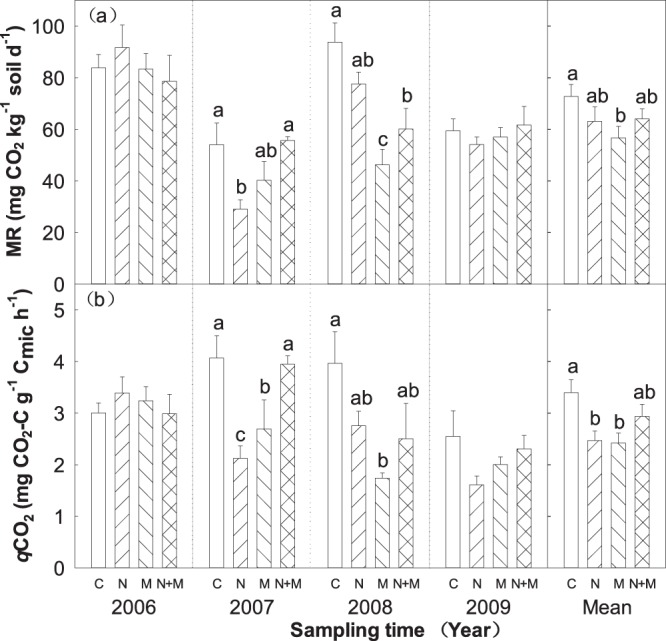
Effects of N addition and mowing on soil microbial respiration (MR) and microbial respiration quotient (*q*CO_2_). Data are represented as the means ± SE (standard error). Significant differences are denoted by different letters (*P* < 0.05). C: control; N: nitrogen addition; M: mowing; N + M: nitrogen addition plus mowing.

**Figure 5:**
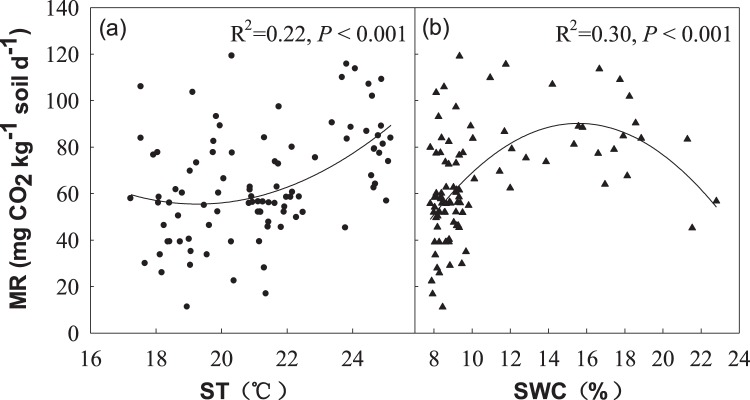
Relationships of soil microbial respiration (MR) with soil temperature (ST) and soil water content (SWC).

There were significant interannual variation in soil microbial respiration (MR) and metabolic quotient (*q*CO_2_) across 4 years (Table 1). N, M and N + M treatments had no significant effect on soil MR and *q*CO_2_ in 2006 and 2009 (Fig. 4). However, soil MR and *q*CO_2_ in C treatment were significantly greater than N + M treatment, M treatment, and N treatment in 2007, seperately (Fig. 4). In addition, soil MR and *q*CO_2_ in C treatment were significant higher than N and N + M treatments and M treatment in 2008 (Fig. 4). In average, soil MR in C treatment was greater than N and N + M treatments, which were significantly greater than mowing across 4 years (Fig. 4a). Meanwhile, soil *q*CO_2_ in C treatment was greater than N + M, which were significant greater than N and M across 4 years (Fig. 4b). Moreover, we observed a significant correlation between soil MR and ST (R^2^ = 0.22, *P* < 0.001; Fig. 5a) and SWC (R^2^ = 0.30, *P* < 0.001; Fig. 5b), respectively.

### N addition and mowing effect on soil net ammonification, nitrification and N mineralization rates

**Figure 6:**
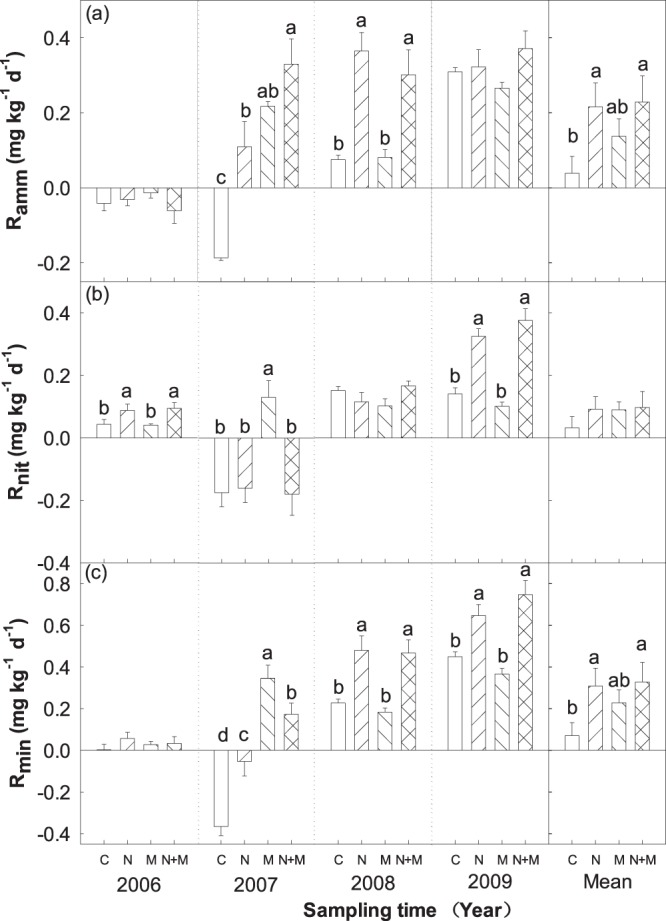
Effects of N addition and mowing on soil ammonification rate (R_amm_), nitrification rate (R_nit_) and net N mineralization rate (R_min_). Data are represented as the means ± SE (standard error). Significant differences are denoted by different letters (*P* < 0.05). C: control; N: nitrogen addition; M: mowing; N + M: nitrogen addition plus mowing.

Significant interannual variation in soil net ammonification, nitrification and N mineralization rates (R_amm_, R_nit_, R_min_) were detected (Table 1). N, M and N + M treatments had no significant effect on soil R_amm_ in 2006 and 2009 (Fig. 6a). However, soil R_amm_ in N + M was greater than M treatment, N treatment and control, respectively (Fig. 6a). N and N + M treatments in soil R_amm_ were significantly higher than that in M and C treatments in 2008 (Fig. 6a). In average, N and N + M treatments in soil R_amm_ were higher than control and M treatment across 4 years (Fig. 6a).

Soil R_nit_ in N and N + M treatments were significantly higher than C and M treatment in 2006 and 2009, seperately (Fig. 6b). Soil R_nit_ in M treatment was significantly higher than C, N and N + M treatments in 2007 (Fig. 6b). However, N, M and N + M treatments had no significant effects on soil R_nit_ in 2008 (Fig. 6b). Compared with control, soil R_nit_ in N, M and N + M treatments were slightly greater across 4 years (Fig. 6b).

**Figure 7:**
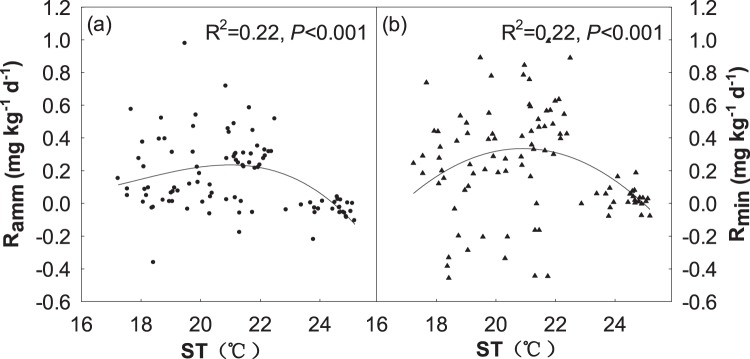
Relationships of soil ammonification rate (R_amm_) and net N mineralization rate (R_min_) with soil temperature (ST).

**Figure 8:**
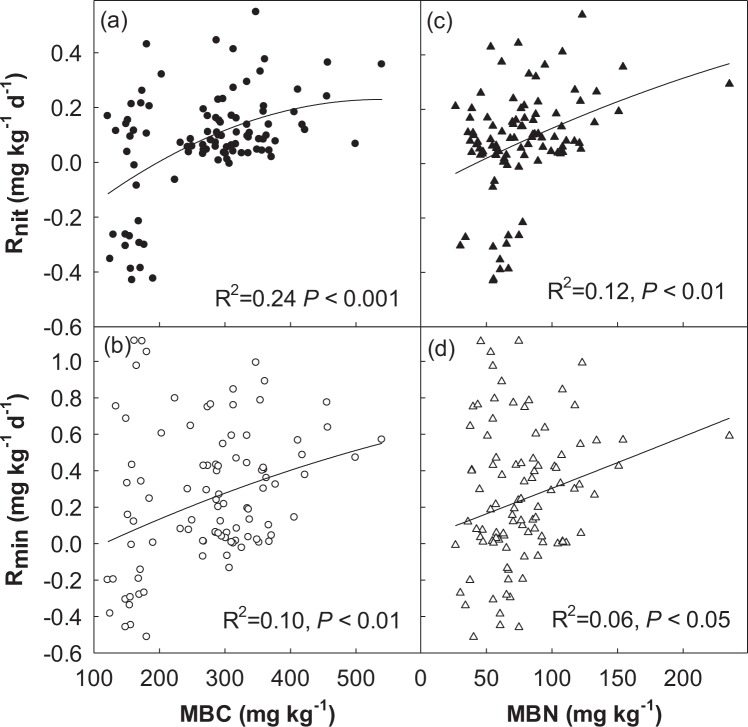
Relationships of soil nitrification rate (R_nit_) and net N mineralization rate (R_min_) with soil microbial biomass carbon (MBC) and microbial biomass nitrogen (MBN).

N, M and N + M treatments had no significant effect on soil R_min_ in 2006 (Fig. 6c). Soil R_min_ in M treatment was significantly greater than that in N + M treatment, N treatment and control in 2007 (Fig. 6c). Soil R_min_ in N and N + M treatments were significantly greater than C and M treatments in 2008 and 2009 (Fig. 6c). In average, N and N + M treatments in soil R_min_ were greater than M treatment, which were significantly greater than C treatment across 4 years (Fig. 6c). Research suggested that ST, SWC and microbial biomass have been well documented to affect soil N mineralization rate in various ecosystems. It was supported by our observations that soil N mineralization rate was strongly correlated with ST, MBC and MBN at both temporal and spatial. For example, soil R_amm_ and R_min_ had a significant non-linear correlation with ST (R^2^ = 0.22, *P* < 0.001; R^2^ = 0.22, *P* < 0.001, respectively; Fig. 7). Meanwhile, we observed a significant non-linear relationships between soil R_nit_ and MBC (R^2^ = 0.24, *P* < 0.001; Fig. 8a) and MBN (R^2^ = 0.12, *P* < 0.01; Fig. 8c), we also observed a significant non-linear relationships between soil R_min_ and MBC (R^2^ = 0.10, *P* < 0.01; Fig. 8b) and MBN (R^2^ = 06, *P* < 0.05; Fig. 8d).

## Discussion

### Effects of N addition on microbial N transformation and microbial activity

The microbes and plant growth usually limited by available N in the N-limited temperate grassland. Thus, microbes and plant growth could be stimulated by increasing soil N content^6,29,30^. We found that N treatment was significantly increased soil ammonification rate (Ramm) and net N mineralization rate (Rnit), and slightly increased nitrification rate (Rnit) across 4 years in our field experiment, which were consisted with the results of the majority of previous research. For example, Dijkstra *et al*.^31^ found that N fertilizer stimulated net N mineralization in a grassland ecosystem in Central America. Even though soil temperature (ST) and soil water content (SWC) were important factors in controlling the process of N transformations. However, our results suggest that N treatment had no significant effects on ST and SWC in this area of grassland, which suggests that ST and SWC could not be well explained the changes of soil net N transformation rate in this area.

Microbial biomass has been found to be a good indicator of N transformation processes at laboratory and field experiments^32^. Nitrogen treatment can increase or decrease microbial activity, yet it is not clear if these impacts are a direct function of the increase in N availability or a result of indirect effects of the fertilizer inputs on other soil chemical characteristics^7,33^. Results from our study showed that the microbial biomass and respiration had significantly changed after 4 years of N addition. In our study, N treatment had no significant effect on microbial biomass C (MBC) and the ratio of MBC/MBN. However, besides the data of 2008 still did not reveal a stimulating effect of N treatment on microbial biomass N (MBN), all other years showed such a stimulation effect. Meanwhile, N treatment had no effect on soil net N transformation rate in 2006, but increased soil net N transformation rate in other years. Accordingly, a stimulating effect of N treatment on microbial N transformation and MBN could be demonstrated. Nitrogen addition increased soil inorganic N pool and stimulated the decomposition of soil organic matter by the microbe-enzyme system. Thus, the stimulation of soil microbial N turnover following N addition is probably not only a response to increased soil N availability but also a feedback to N-induced increases in plant growth^34^. These results were consistent with previous studies, soil microorganisms immobilized a higher proportion of mineralized N after 8 years of N fertilization, and that was likely due to greater specific activity and turnover of microbial biomass in N treatment^35^. Our results also showed that N treatment slightly decreased microbial respiration (MR) and significantly reduced respiratory quotient (*q*CO_2_). Previous study indicated that N addition increased the available N content in grassland soil, and significantly increased the labile C sources in the topsoil. Meanwhile, N addition did not only increase the level of available N but also decreased soil pH, which may be the major reason for no significant effect on soil MR but significantly increased soil N transformation rate under N addition treatments.

### Effects of mowing on microbial N transformation and microbial activity

Mowing (M) is an important land-use type in grassland ecosystem, it could reduce C inputs to soil and also remove a large amount of N out of grassland ecosystem, reduced the availability of decomposable substrates for soil microorganisms^36^. Many studies have shown that mowing promoted the aboveground and belowground parts of the plant growth and root secretion, affected the process of soil N transformation^37^. However, we found that mowing had no significant effects on soil microbial N transformation across 4 years in our field experiment. Consistent with our results, Maron and Jefferies^38^ examined the effects of mowing on N rich species richness, grassland yield and N retention, across 5 years spring mowing did not cause changes in net N mineralization rate. Two possible reasons could help to explain the little changes in soil N transformations under mowing observed in this study. Firstly, the possible reason why N transformation did not change was that soil temperature and moisture unaltered. Temporal variations in soil temperature and soil moisture can significantly drive microbial activities and N transformations in grassland ecosystems^39^. For example, Wang *et al*.^40^ found in a laboratory study that N transformation and microbial activity in typical temperate steppe near our field experiment site was driven by changes in soil temperature and moisture. The study about the effects of mowing on soil N mineralization by Snapp and Borden^41^ who found that the response of soil microbes to mowing might be affected by soil water content. However, mowing had no significant effect on soil temperature and water content in our study site. The mechanism of mowing effects on soil microbial N transformations were controlled by the complex interaction of changing soil microclimate. Another reason was that although the supply of C substrate to soil microorganisms decreased, but soil microbial activity might be unaltered. Our study showed that mowing had no significant effect on soil microbial biomass and slightly increased the ratio of MBC/MBN. Although the microbial community has changed, but the microbial biomass had no significant changes across 4 years. In addition, the supply of C substrate to soil microorganisms decreased during the same period in this study^11^. Previous studies suggested that the removal of plant aboveground biomass and litter with a large C:N ratio facilitated rapid N cycling by limiting carbon input to the soil, thus maintaining rapid N transformation in the microbial community^24^. Inconsistent with our research, some studies suggested that mowing reduced the ground organic carbon input to soil and reduced the energy for microbial activities, reduced the microbial biomass C and N ratio, thereby decreased the rate of N transformation^42–44^.

Mowing significantly decreases soil MR and *q*CO_2_. However, both increased^45^ and reduced^46,47^ soil temperature and moisture have been reported in response to mowing in the previous studies. Consistent with our results, Jia *et al*.^48^ and Han *et al*.^11^ found no response of soil respiration to mowing due to the unaltered plant growth and soil moisture. Given the strong regulation of soil water availability on root and microbial activity^49–51^, but the response of soil MR to mowing observed in this study might not be accounted for the unchanged ST and SWC. Mowing was also expected to affect soil MR through its influence on C substrate^36^. Previous studies suggested that a smaller metabolic quotient indicates a more efficient use of substrates by microorganisms, where a greater fraction of substrate C was incorporated into microbial biomass and less C per unit biomass was lost through respiration^24^. In addition, mowing reduced the supply of C and N substrate to soil microorganisms during the same period in this study^11^. These facts prove that mowing reduced the availability of decomposable substrates for soil microorganisms, and reduced C inputs to soil and lead to a large amount of N loss, caused substrate limitation to microorganisms^37,47^. Therefore, C and N substrate were the main factor affecting soil MR in this study.

### Interaction effects of N addition and mowing on microbial N transformation and microbial activity

Generally, we hold the opinion that mowing may mask the N fertilizer effects on soil microbial communities by reducing plant litter input and root exudation and thus reducing soil microbial biomass and activities. However, soil microorganisms (ST and SWC) and the microbial variables (MBC, MBN and MBC/MBN) had no significant changes in our study. Meanwhile, N + M treatments had no significant effect on MR and *q*CO_2_. These results were in line with the studies by Johnson *et al*.^52^ and Stark and Kytöviita^53^, who found that fertilizer increased *in situ* soil respiration (plant plus microbial respiration) but did not affect the MR measured in the laboratory from sieved soil samples. Previous studies have shown that mowing probably resulted in a depletion of labile C sources in the topsoil and, together with increased N uptake by the plants as a response to mowing, soil microbes may have become C as well as N limited^23^. However, previous research found that N addition with or without mowing in grassland increased soil inorganic N, which alleviated the limitation for plant growth and enhanced its growth and photosynthetic capacity^24^. Our study found that N + M treatment was significantly increased the soil inorganic N pool and soil total organic C content, but had no significant effect on microbial biomass and activities. This could explain our finding that MR did not increase.

Mowing may significantly influence the direct impacts of N addition on the soil environment and on soil microbial N transformation responses to N addition^54^. We found that 4 years of continuous N application with mowing led to significant increase in R_amm_ and R_min_ but had no significant effects on R_nit_. Consistent with our study, Wang *et al*.^24^ found that mowing decreased SWC and increased ST at the same time, leading to low microbial activity, soil N mineralization rate decreased, but N + M treatment was significantly increased soil microbial N mineralization rate. Although N + M treatment had no significant effect on microbial biomass and activities, but significantly increased the soil inorganic N pool and soil total organic C content (Unpublished data). Results from our study indicate that 4 years of continuous N fertilizer application with mowing or without mowing led to significant changes in microbial N transformation in the abandoned grassland ecosystem where we studied, resulting in an increase in rates of net N mineralization. These conclusions confirmed the predictions of our theoretical hypothesis.

## Conclusions

Nitrogen addition and mowing simultaneously increased net N mineralization rate in an abandoned grassland over a 4-year period experiment (2006–2009). Mowing slightly increased soil net N mineralization rates. However, N addition with or without mowing led to significant effects on microbial N transformation. In addition, N addition and Mowing treatments were decreased microbial biomass and microbial respiration according to 4 years, while N + M treatment had no significant effects on microbial biomass and microbial respiration. From our short time experiment, we made a conclusion that N + M treatment did not weaken soil net N mineralization rates in a semi-arid grassland of Northern China^55,56^.
